# Ultrastructural and molecular study of the microsporidian *Toguebayea baccigeri* n. gen., n. sp., a hyperparasite of the digenean trematode *Bacciger israelensis* (Faustulidae), a parasite of *Boops boops* (Teleostei, Sparidae)

**DOI:** 10.1051/parasite/2022007

**Published:** 2022-02-01

**Authors:** Jordi Miquel, Hichem Kacem, Edgar Baz-González, Pilar Foronda, Bernard Marchand

**Affiliations:** 1 Secció de Parasitologia, Departament de Biologia, Sanitat i Medi Ambient, Facultat de Farmàcia i Ciències de l’Alimentació, Universitat de Barcelona Av. Joan XXIII, sn 08028 Barcelona Spain; 2 Institut de Recerca de la Biodiversitat (IRBio), Universitat de Barcelona Av. Diagonal, 645 08028 Barcelona Spain; 3 Laboratoire de Biodiversité Marine et Environnement, Département des Sciences de la Vie, Faculté des Sciences de Sfax, Université de Sfax BP 1171 3000 Sfax Tunisia; 4 Instituto Universitario de Enfermedades Tropicales y Salud Pública de Canarias, Universidad de La Laguna 38203 La Laguna Canary Islands, Spain; 5 Departmento de Obstetricia y Ginecología, Pediatría, Medicina Preventiva y Salud Pública, Toxicología, Medicina Legal y Forense y Parasitología, Facultad de Farmacia, Universidad de La Laguna 38203 La Laguna Canary Islands, Spain; 6 CNRS, UMR SPE 6134, Université de Corse Pasquale Paoli, Projet GEM 20250 Corte France

**Keywords:** Hyperparasite, *Toguebayea baccigeri* n. gen. n. sp., Microsporidia, *Bacciger israelensis*, Digenea, Phylogenetic analysis

## Abstract

A new microsporidian *Toguebayea baccigeri* n. gen., n. sp., hyperparasite of *Bacciger israelensis* (Digenea, Faustulidae), parasite of *Boops boops* (Teleostei, Sparidae) is described by means of transmission electron microscopy. The phylogenetic analysis, based on the SSU rDNA gene, places the new species in the clade containing mainly crustacean-infecting microsporidia of the genus *Cucumispora*, within superclade V (Marinosporidia) *sensu* Vossbrinck et al., 2014. Mature spores of *T. baccigeri* are ovoid, uninucleated and measure 2.5 × 1.4 μm. The number of coils of the polar tube is 8–10. The polaroplast is composed of an external lamellar part and an internal vesicular or granular part. The main differences that distinguish the new genus and new species from the closely related microsporidia include hyperparasitism in a digenean host infecting a marine fish, the geographic distribution (coast of Tunisia), presence of one nucleus at all developmental stages, disporoblastic sporogony, and the absence of sporophorous vacuoles.

## Abstract (fr)

Une nouvelle microsporidie *Toguebayea baccigeri* n. gen., n. sp., hyperparasite de *Bacciger israelensis* (Digenea, Faustulidae), parasite de *Boops boops* (Teleostei, Sparidae) est décrite en microscopie électronique à transmission. L’analyse phylogénétique, basée sur l’étude du gène SSU rDNA, place la nouvelle espèce dans le clade qui contient notamment les microsporidies parasites de crustacés du genre *Cucumispora*, dans le superclade V (Marinosporidia) *sensu* Vossbrinck et al, 2014. Les spores mûres de *T. baccigeri* sont ovoïdes, uninucléées et mesurent 2,5 × 1,4 μm. Le nombre de tours de spire décrits par le tube polaire est de 8 à 10. Le polaroplaste est composé d’une partie externe lamellaire et d’une partie interne vésiculeuse ou granuleuse. Les principales différences entre le nouveau genre et la nouvelle espèce et les microsporidies les plus proches sont : l’hyperparasitisme d’un digène parasite d’un poisson marin, la localisation géographique (côtes de la Tunisie), et certains aspects du développement, notamment la présence d’un seul noyau à tous les stades de développement, la sporogonie disporoblastique et l’absence de vacuoles sporophores.

## Introduction

Microsporidia are unicellular organisms and obligate intracellular parasites of numerous animal phyla, including more than 1300 species belonging to more than 220 genera. Some species can infect humans, causing microsporidiosis particularly in immunosuppressed people [7, 11]. Although microsporidians were once considered protozoans [15], presently, they are included in the kingdom Fungi Moore, 1980, phylum Rozellomycota Doweld, 2013 and class Microsporidea Corliss & Levine, 1963 [5, 21]. Moreover, several microsporidia have been recorded as hyperparasites, particularly in digenean platyhelminths. According to Sokolova and Overstreet [13], microsporidia parasitizing digeneans are restricted to five genera, namely *Microsporidium* Balbiani, 1884, *Nosema* Nägeli, 1857, *Ovipleistophora* Pekkarinen, Lom & Nilsen, 2002, *Pleistophora* Gurley, 1893 and *Unikaryon* Canning, Lai & Lie, 1974. Microsporidians grow and reproduce inside their hosts cells; their lifecycles follow two phases of development, merogony or schizogony and sporogony. Merogony is a proliferation phase producing meronts, and sporogony leads to the formation of sporoblasts that undergo morphogenesis into spores. The spores are the only developmental stage that is present outside of the host [3, 16, 22].

Among microsporidia, Clade V [25] comprises mostly microsporidia infecting fishes and crustaceans from marine and freshwater habitats. Three microsporidia reported as hyperparasites are included in this clade: *Unikaryon legeri* Canning & Nicholas, 1974, *Unikaryon panopei* Sokolova, Overstreet, Heard & Isakova, 2021 and *Hyperspora aquatica* Stentiford, Ramilo, Abollo, Kerr, Bateman, Feist, Bass & Villalba, 2017 [11, 14, 18].

In the present study, we describe a new genus and a new species by means of transmission electron microscopy, *Toguebayea baccigeri* n. gen., n. sp., hyperparasite of *Bacciger israelensis* Fischthal, 1980 (Digenea, Faustulidae), a parasite of the bogue *Boops boops* (Linnaeus, 1758) (Teleostei, Sparidae) from the coast of Tunisia. We provide an SSU rDNA-based phylogenetic analysis of relationships of *T. baccigeri* n. gen., n. sp. with related species/genera.

## Materials and methods

### Specimens

A total of 73 live adult specimens of *Bacciger israelensis* Fischthal, 1980 (Digenea, Faustulidae) were gathered from the pyloric caeca of 89 bogues *Boops boops* (Linnaeus, 1758) (Teleostei, Sparidae) caught by means of artisanal fishing in March 2019 and January 2021 in the Mediterranean Sea (35° 22′ N 11° 04′ E and 35° 19′ N 11° 05′ E), off Salakta (Tunisia). Prevalence of *B. israelensis* was 34.8% and mean intensity was 2.35 (with a range of 1–7). Adult digeneans were identified according to the available literature [1, 4, 6]. While studying the ultrastructure of *B. israelensis*, we came across infection with microsporidia. Three flukes among six examined were infected with microsporidia.

### Transmission electron microscopy

Adult live flukes were rinsed with a 0.9% NaCl solution and fixed in cold (4 °C) 2.5% glutaraldehyde in a 0.1 M sodium cacodylate buffer at pH 7.4 for 2 h, rinsed in 0.1 M sodium cacodylate buffer at pH 7.4, post-fixed in cold (4 °C) 1% osmium tetroxide with 0.9% potassium ferricyanide in the same buffer for 1 h, rinsed in Milli-Q water (Millipore Gradient A10), dehydrated in graded ethanol series and propylene oxide, embedded in Spurr’s resin and polymerized at 60 °C for 72 h. Semithin sections (500 nm thick) were obtained using a Reichert-Jung Ultracut E ultramicrotome and stained with 1% methylene-blue with 1% borax. Ultrathin sections (60–90 nm thick) containing the excretory vesicle were prepared with the same ultramicrotome. Sections were placed on copper grids (200 μm mesh size) and double-stained with uranyl acetate and lead citrate according to the Reynolds procedure [12]. Stained ultrathin sections were examined in a JEOL 1010 transmission electron microscope operated at an accelerating voltage of 80 kV, equipped with a CCD camera (Gatan Orius model SC1000A1) in the “Centres Científics i Tecnològics” of the University of Barcelona (CCiTUB). All microsporidian measurements were obtained using the Gatan Microscopy Suite Digital Micrograph version 2.11.1404.0 coupled to the CCD camera.

### Molecular analyses and phylogenetic tree

Total DNA was isolated from 17 digeneans (fixed in absolute ethanol) obtained from eight fishes. For DNA isolation, a commercial FastDNA^®^ Spin Kit for Soil (MPBiomedicals, Solon, OH, USA) was used following the manufacturer’s instructions, with the homogenizer FastPrep-24™ 5G (MP Biomedicals) as cellular disruptor.

A fragment of the SSU rDNA of the microsporidia was amplified by PCR targeting a 1200 bp region following Sokolova et al. [14] with the primer V1f and primer 1492r [23]. The reaction mixture contained 1U Taq DNA polymerase, 1 μL of each primer (10 μM), 2.5 μL of dNTPs mix (200 μM), 2.5 μL MgCl_2_ (25 mM), 2.5 μL 10× buffer (15 mM Mg^2+^), 5 μL of DNA template and water to a total volume of 25 μL. The cycling conditions were initial denaturation of 95 °C for 5 min followed by 35 cycles of 95 °C for 30 s, 45 °C for 60 s at suitable temperature, and 120 s at 72 °C, followed by a final extension step at 72 °C for 10 min. The PCR reactions were performed in an XP Cycler (Bioer Technology, Hangzhou, PR China) thermocycler. PCR products were resolved on 2% agarose gels. The desired size band was cut from the gel and purified with an EZNA Gel Extraction Kit (Omega Bio-Tek, Norcross, GA, USA), following the manufacturer’s recommendations. The purified PCR products were sequenced at Macrogen Europe (Madrid, Spain) with primers V1f, 530r, 530f, 1061f, and 1492r [23, 26].

The obtained nucleotide sequences were edited with the MEGA X program [8] and subsequently aligned with other microsporidian sequences using the ClustalW program included in MEGA X. Minor corrections, to increase the aligned sequence similarity and improve the inferences on any positional homology, were then made by hand. A BLAST search was carried out in order to elucidate any homologies or similarities with the sequences previously published in the GenBank database (Supplementary material Table S1).

**Figure 1:**
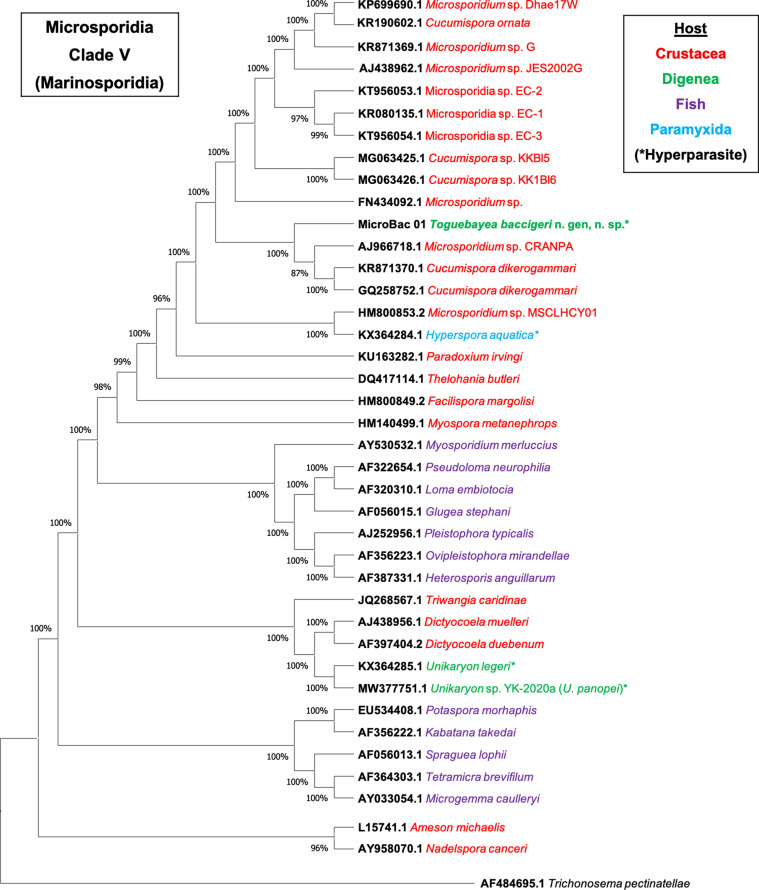
Maximum Likelihood phylogenetic analysis of Microsporidia sequences based on the SSU rDNA gene. *Trichonosema pectinatellae* (AF484695.1) was used as the outgroup. MicroBac01 corresponds to the sequence obtained for *Toguebayea baccigeri* n. gen., n. sp.

The molecular identification was achieved by phylogenetic analysis through the Neighbour-Joining distance method with the Kimura 2-parameter estimate [9] with at least 1000 bootstrap replications (Supplementary material Figure S1); and the Maximum Likelihood method with the Tamura-Nei model [20] in MEGA X, using the sequence of *Trichonosema pectinatellae* Canning, Refardt, Vossbrinck, Okamura & Curry, 2002 (AF484695.1) as the outgroup (Fig. 1). Distance estimation was carried out in MEGA X using the p-distance model distance matrix for transitions and transversions (Kimura 2-parameter), with partial deletion of position with gaps or missing data (Supplementary material Table S2). The nucleotide sequence obtained in this work was submitted to the GenBank database under accession no. MZ413057.

## Results

### Genus *Toguebayea* n. gen.

urn:lsid:zoobank.org:act:53B8CC8E-D5AB-46FA-A318-28BADF598DA2

*Definition*: Hyperparasite of *Bacciger israelensis* (Digenea: Faustulidae) parasite from pyloric caeca of the fish *Boops boops* (L., 1758) (Teleostei, Sparidae). Parasite life stages infecting the epithelium of the excretory vesicle of the digenean *B. israelensis*. All developmental stages in contact with host cell cytoplasm and lacking sporophorous vacuoles. Lifecycle is unikaryotic and develops from uninucleated to binucleated meronts and subsequent cell division prior to sporogony. Disporoblastic sporogony generates uninucleated sporonts and later uninucleated sporoblasts. Sporoblasts mature to spores, which continue to occupy the cytoplasm of the epithelial cells of the excretory vesicle. Spores ovoid and uninucleated, approximately 2.5 × 1.4 μm.

*Type species*: *Toguebayea baccigeri* n. gen., n. sp.

### *Toguebayea baccigeri* n. gen., n. sp.

urn:lsid:zoobank.org:act:B182EACC-4E16-43FC-8576-59F89824592E

*Description*: As for the genus.

*Diagnosis*: Presence of a microsporidian hyperparasite with descriptive features of the genus in the cytoplasm of epithelial cells of the excretory vesicle of *B. israelensis* infecting the pyloric caeca of *B. boops*. Diagnosis of morphological features by TEM as described herein. Spores ovoid and uninucleated, approximately 2.5 × 1.4 μm; exospore around 40 nm wide; endospore around 50 nm wide and about 5 nm wide at the level of the apical part near the anchoring disk; polar sac 30–40 nm wide; isofilar polar tube with 8–10 coils arranged in one or two rows, with a diameter of 95–115 nm, composed of alternate concentric electron-dense and electron lucent layers with a central tubular element 8 nm wide; manubrium around 115 nm wide and 850 nm long; well-developed polaroplast lamellar in the external part and granular in the internal part; nucleus around 650 × 400 nm; posterior vacuole irregularly round with a diameter around 375 nm. Nucleic acid-based diagnosis via PCR ampliﬁcation, analysis of the deﬁned SSU rDNA gene sequence (MZ413057) and comparison with GenBank sequences.

*Type host*: Hyperparasite of *Bacciger israelensis* Fischthal, 1980 (Digenea, Faustulidae) parasite from the pyloric caeca of *Boops boops* (Linnaeus, 1758) (Teleostei, Sparidae).

*Type locality*: Mediterranean Sea, off Salakta (35° 22′ N 11° 04′ E and 35° 19′ N 11° 05′ E) (Tunisia).

*Site of infection*: Epithelium of the excretory vesicle.

*Type material*: Two slides with methylene-blue stained semithin sections of two *Bacciger israelensis* specimens (TEM blocks 069/19 and 070/19) infected with *Toguebayea baccigeri* n. gen., n. sp., ex. *Boops boops* (no. 2019032205) off Salakta (Tunisia), 22 March 2019 are deposited in the “Muséum National d’Histoire Naturelle” (Paris, France) – accession nos. MNHN-IR-2021-1 and MNHN-IR-2021-2. TEM blocks of infected *B. israelensis* (accession nos. 069/19, 070/19 and others) are filed in the Section of Parasitology, Department of Biology, Health and Environment, University of Barcelona, Spain (JM’s collection of TEM blocks of parasitic Platyhelminthes).

*SSU rDNA sequence*: GenBank, under accession no. MZ413057.

*Etymology*: The generic name refers to Prof. Bhen Sikina Toguebaye from the University Cheikh Anta Diop of Dakar (Senegal), a specialist in microsporidians. The specific epithet refers to the genus of the infected host, *Bacciger israelensis*.

#### Description of *Toguebayea baccigeri* n. gen., n. sp. (Figs. 2–10; Table 1)

**Figure 2:**
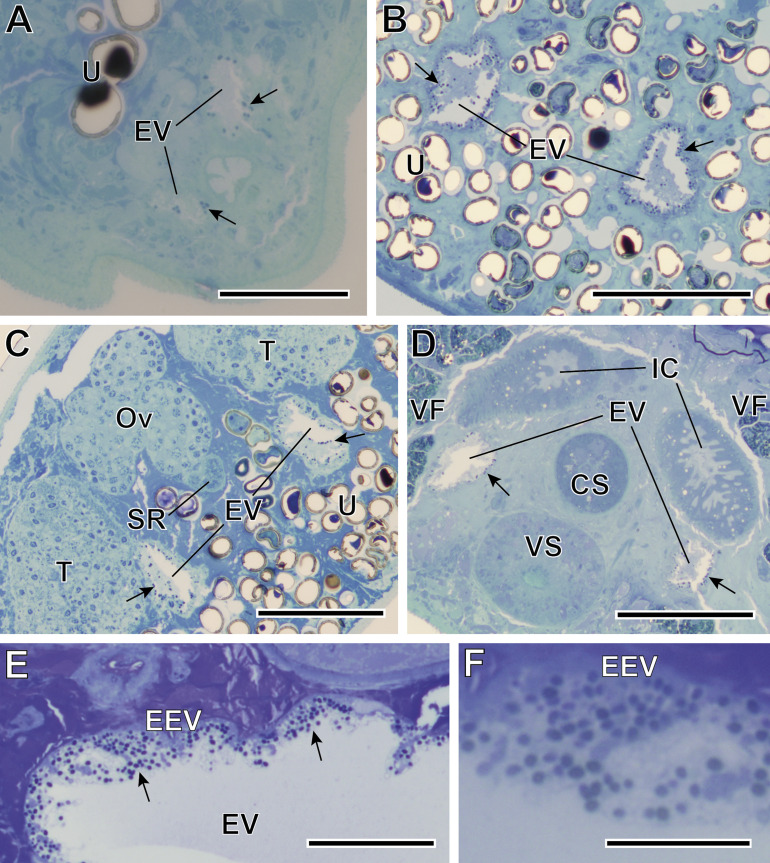
Semithin sections of *Bacciger israelensis* stained with methylene-blue at different levels showing the presence of many spores (black arrows). (A–B) Sections at 35 and 155 μm from the posterior extremity of the digenean showing the two arms of the Y-shaped excretory vesicle (EV); (C) Section at 210 μm from the posterior extremity at the level of genitalia, showing both testes (T) and the characteristic trilobed ovary (Ov); (D) Section at 310 μm from the posterior extremity at the level of the ventral sucker (VS) and the cirrus sac (CS); (E) Detail of the excretory vesicle at 150 μm from the anterior extremity showing numerous *T. baccigeri* n. gen., n. sp. in its epithelium (EEV); (F) Enlarged detail of the epithelium of the excretory vesicle. IC, intestinal caeca; SR, seminal receptacle; U, uterus; VF, vitelline follicles. Scale bars: A, E = 50 μm; B–D = 100 μm; F = 20 μm.

**Figure 3:**
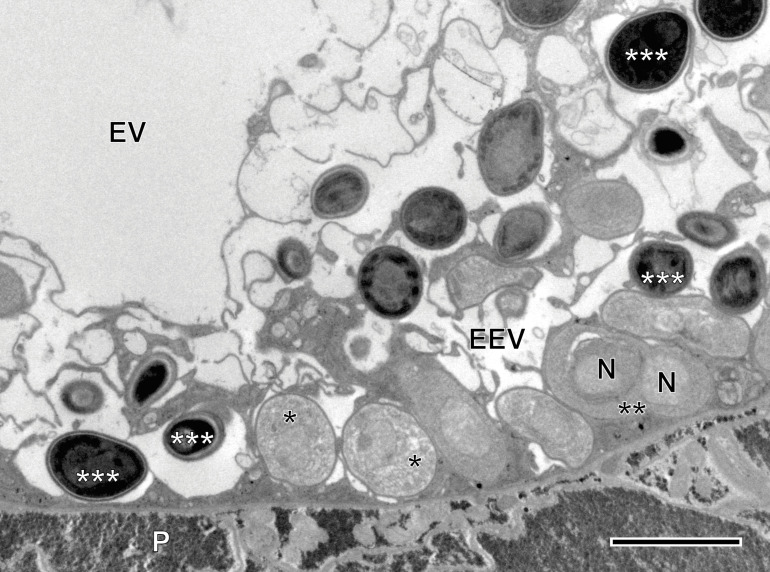
TEM micrograph showing different development stages of *Toguebayea baccigeri* n. gen., n. sp. in the epithelium of the host’s excretory vesicle (EEV). *, merozoites; **, dividing meront; ***, mature spores; EV, excretory vesicle; N, nucleus; P, parenchyma. Scale bar = 2.5 μm.

Microsporidian infection was found in adults of *Bacciger israelensis*, a digenean parasite of the bogue *Boops boops* caught off the coast of Tunisia. The pathogen was present along the entire length of the hosts excretory vesicle epithelium (Figs. 2 and 3). There was no evidence of spore groups enclosed in sporophorous vacuoles. Each developmental stage occurred in direct contact with the cytoplasm of the host cell and had only one nucleus (Figs. 3 and 5–8).

**Figure 4:**
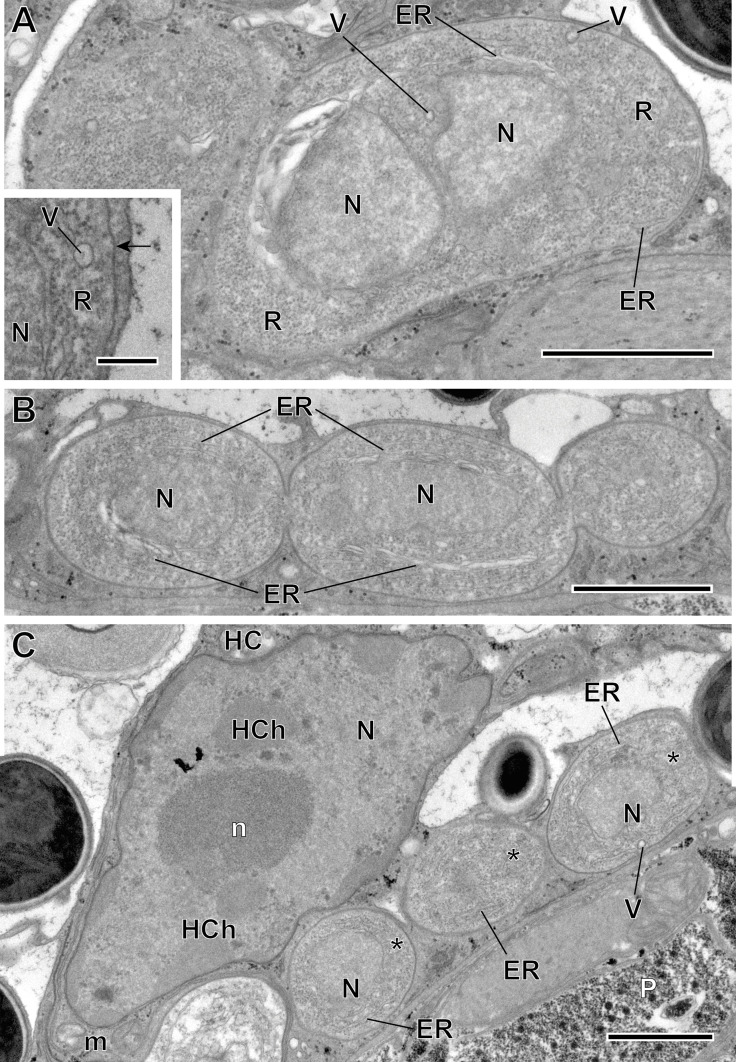
Merogonic stages of *Toguebayea baccigeri* n. gen., n. sp. (A–B) Dividing meronts; (C) Merozoites (*) in a host cell (HC); (inset) Detail at high magnification of the meront plasma membrane (black arrow). ER, endoplasmic reticulum; HCh, heterochromatin cluster; m, mitochondrion; N, nucleus; n, nucleolus; P, parenchyma; R, ribosomes; V, vacuoles. Scale bars: A–C = 1 μm; inset = 0.2 μm.

**Figure 5:**
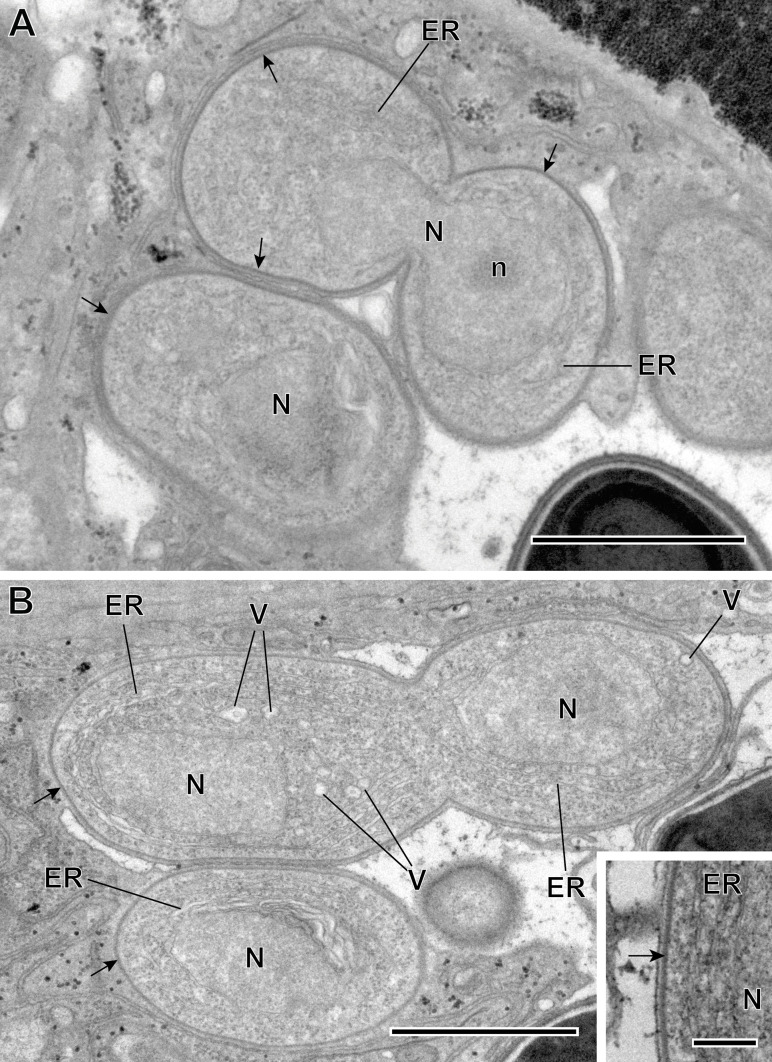
(A–B) Sporogonic stage of *Toguebayea baccigeri* n. gen., n. sp. Note the thick membrane of sporonts (black arrows) in comparison with meronts; (inset) Detail at high magnification of the sporont plasma membrane (black arrow). ER, endoplasmic reticulum; N, nucleus; n, nucleolus; V, vacuoles. Scale bars: A–B = 1 μm; inset = 0.2 μm.

**Figure 6:**
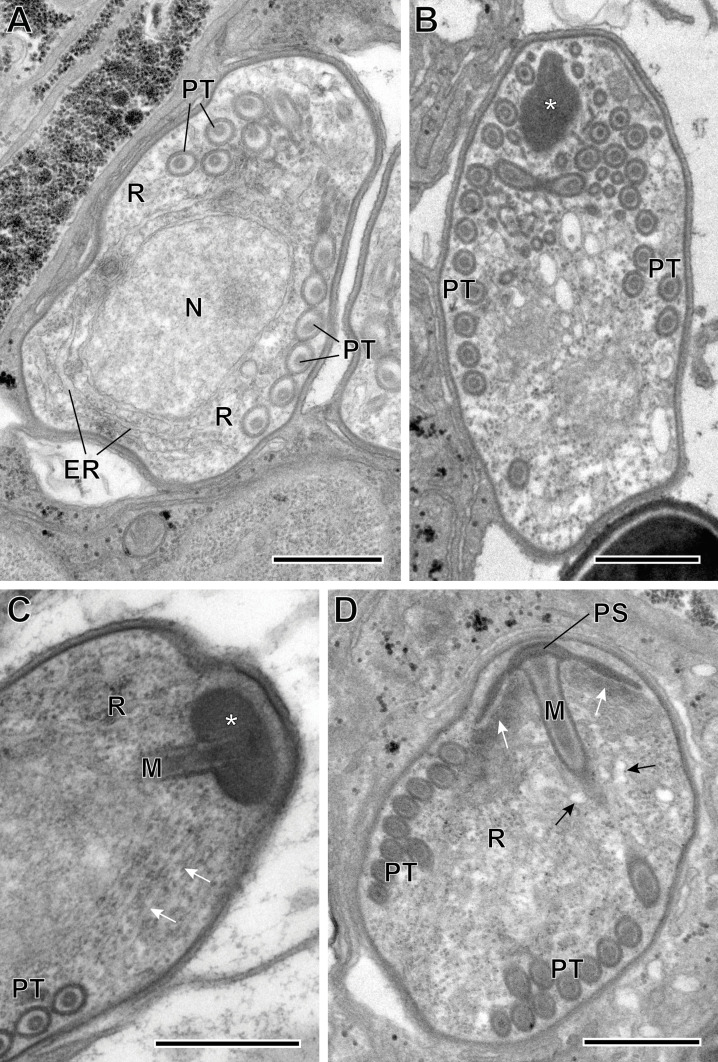
(A–D) Sporoblasts of *Toguebayea baccigeri* n. gen., n. sp*.* Note the flattened saccules (white arrows) and the dilated saccules (black arrows) originating in the lamellar part and the granular or vesicular part of the polaroplast, respectively. *, amorphous electron-dense anterior material; ER, endoplasmic reticulum; M, manubrium; N, nucleus; R, ribosomes; PS, polar sac; PT, polar tube. Scale bars = 0.5 μm.

**Figure 7:**
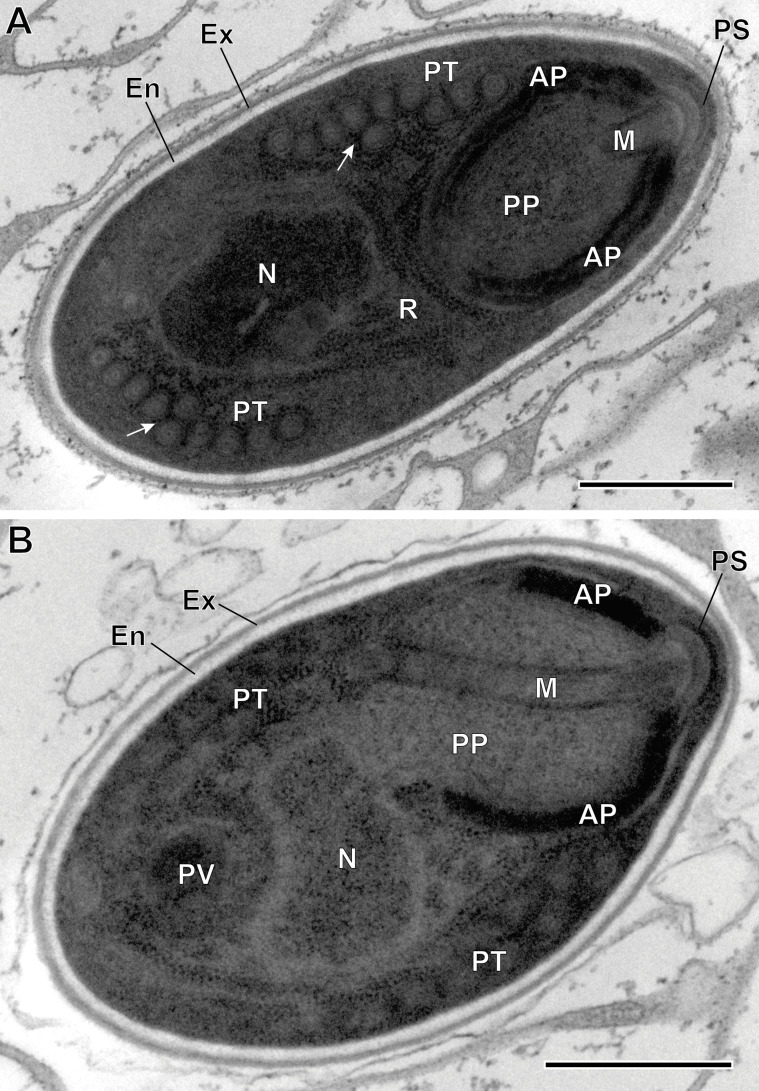
(A–B) Ultrastructural details of mature spores of *Toguebayea baccigeri* n. gen., n. sp observed in longitudinal ultrathin sections. The polar tube (PT) coils are arranged in two rows slightly overlapping each other (white arrows). AP, anterior polaroplast; En, endospore; Ex, exospore; M, manubrium; N, nucleus; PP, posterior polaroplast; PS, polar sac; PV, posterior vacuole; R, ribosomes and polyribosomes. Scale bars = 0.5 μm.

**Figure 8:**
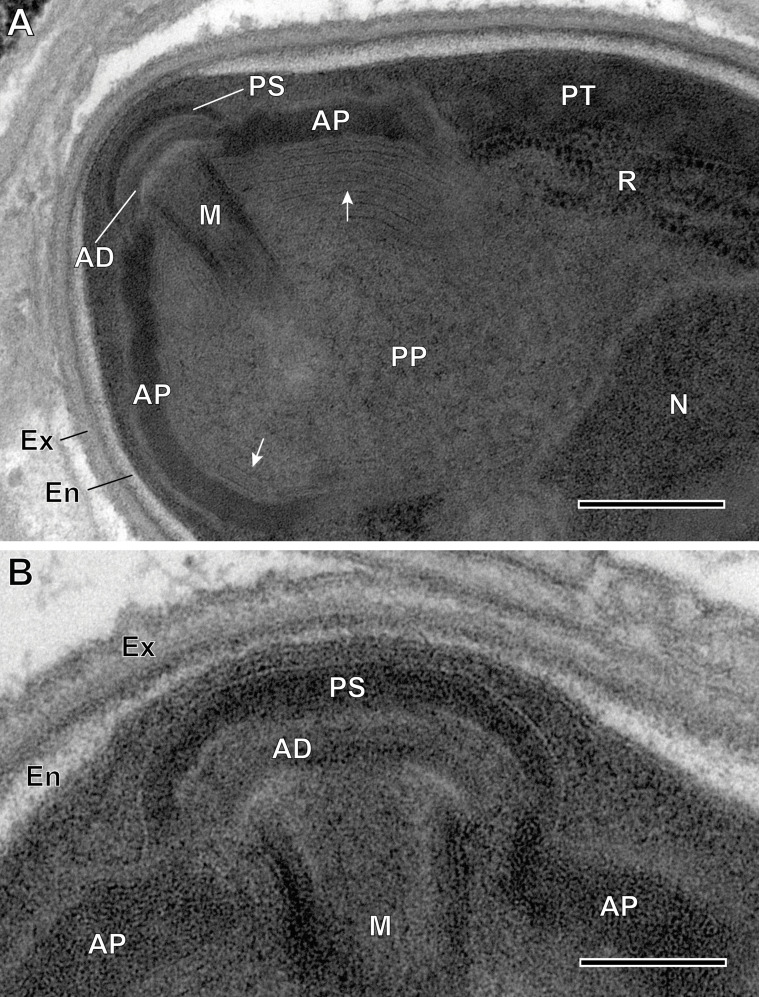
Ultrastructural details of mature spores of *Toguebayea baccigeri* n. gen., n. sp. (A) Anterior region showing the polaroplast with a lamellar external part (white arrows) and a granular internal part; (B) Enlarged detail of the apical part showing the polar sac (PS) and the anchoring disc (AD). AP, anterior polaroplast; En, endospore; Ex, exospore; M, manubrium; N, nucleus; PP, posterior polaroplast; PT, polar tube; R, ribosomes and polyribosomes. Scale bars: A = 0.25 μm; B = 0.1 μm.

**Figure 9:**
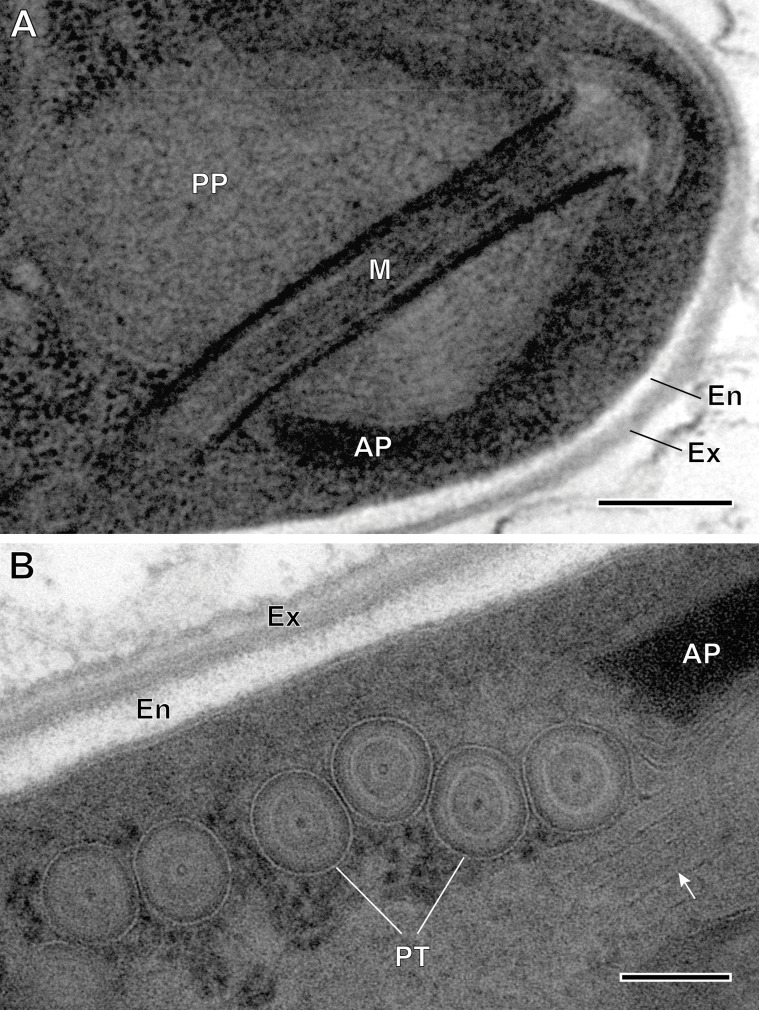
Ultrastructural details of mature spores of *Toguebayea baccigeri* n. gen., n. sp. (A) Detail of the apical part showing the manubrium (M); (B) Detail showing cross-sections of the polar tube (PT) composed of several concentric electron-dense and electron lucent layers surrounding a central tubular structure. Note the lamellar external area (white arrows) of the posterior polaroplast (PP). AP, anterior polaroplast; En, endospore; Ex, exospore. Scale bars: A = 0.2 μm; B = 0.1 μm.

**Figure 10:**
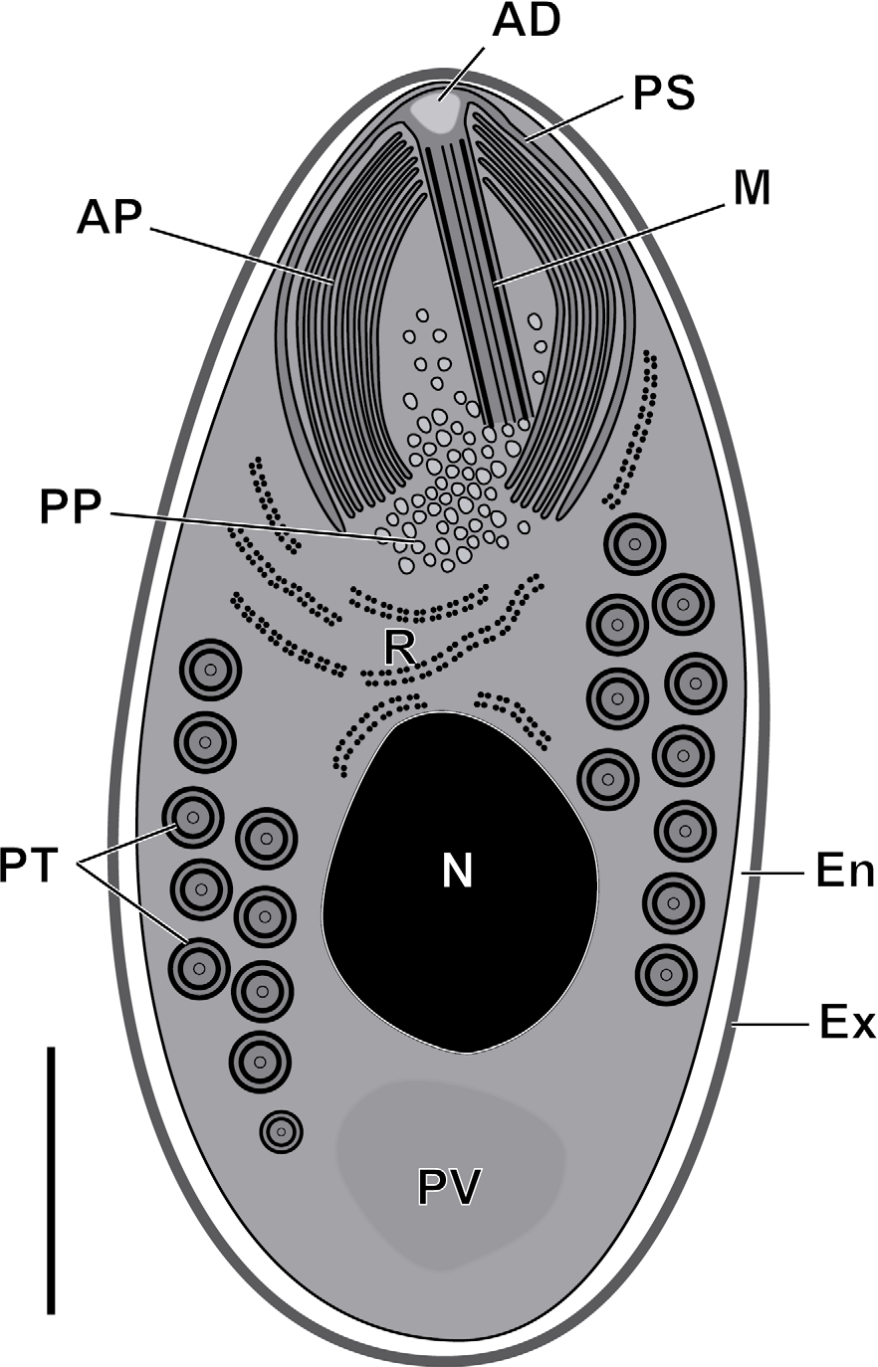
Schematic drawing of the mature spore of *Toguebayea baccigeri* n. gen., n. sp. AD, anchoring disc; AP, anterior polaroplast; En, endospore; Ex, exospore; M, manubrium; N, nucleus; PP, posterior polaroplast; PS, polar sac; PT, polar tube; PV, posterior vacuole; R, ribosomes and polyribosomes. Scale bar = 0.5 μm.

**Table 1:** Some characteristics of certain microsporidian species included in Clade V (Marinosporidia).

Genus [References]	Hosts, lifecycle environment and localities	Main characteristics of spores	GenBank data
*Cucumispora dikerogammari* Ovcharenko, Bacela, Wilkinson, Ironside, Rigaud & Wattier, 2010 [10]	*Dikerogammarus villosus* (Amphipoda), Freshwater, Saone River (France), and Oder River and Zegrzynski Reservoir (Poland)	Elongated, diplokaryotic, 3.82 × 2.21 μm (fresh), 3.74 × 1.91 (Giemsa stained)	GQ258752.1
		Polar tube: isofilar, 6–8 coils in a single layer	
		Absence of SPV	
*Hyperspora aquatica* Stentiford, Ramilo, Abollo, Kerr, Bateman, Feist, Bass & Villalba, 2017 [18]	**Hyperparasite** of *Marteilia collichia* (Paramyxida) in the marine mollusc *Cerastoderma edule*, Ría de Arousa (Spain)	Spherical to ellipsoid, uninucleated, 1.2 × 1.0 μm	KX364284.1
		Polar tube: isofilar, 4 coils in a single layer	
		Absence of SPV	
*Myospora metanephrops* Stentiford, Bateman, Small, Moss, Shields, Reece & Tuck, 2010 [17]	*Metanephrops challengeri* (Decapoda), Marine, Auckland Islands (New Zealand)	Elongated, diplokaryotic, 4.3–6 × 1.7–2.3 μm (glutaraldehyde fixed)	HM140499.1
		Polar tube: isofilar, 11 coils in a single layer	
		Absence of SPV	
*Paradoxium irvingi* Stentiford, Ross, Kerr, Bass & Bateman, 2015 [19]	*Pandalus montagi* (Decapoda), Marine, North Sea (UK)	Oval, uninucleated, 1.93 × 1.07 μm (glutaraldehyde fixed)	KU163282.1
		Polar tube: isofilar, 6–8 coils	
		Absence of SPV	
*Thelohania butleri* Johnston, Vernick & Sprague, 1978 [2]	*Pandalus jordani* (Decapoda), Marine, Vancouver Island (Canada)	Ovoid, uninucleated, 3.95–5.56 × 2.92–3.95 μm (ethanol fixed), 3.25–3.82 × 1.93–2.50 μm (glutaraldehyde fixed)Polar tube: isofilar, 13 coils in two layersPresence of SPV	DQ417114.1
*Toguebayea baccigeri* n. gen., n. sp. [present study]	**Hyperparasite** of adult *Bacciger israelensis* (Digenea, Faustulidae) in the marine fish *Boops boops*, Tunisia	Ovoid, uninucleated, 2.5 × 1.4 μm (glutaraldehyde fixed)	MZ413057
		Polar tube: isofilar, 8–10 coils in one and more frequently two layers	
		Absence of SPV	

SPV, sporophorous vacuole.

##### *Meronts* (Figs. 3 and 4)

Meronts were uninucleated, oval to elongated cells that measure 2–3 μm and were surrounded by a thin plasmalemma (Fig. 4 inset). Their cytoplasm contained a great number of free ribosomes, a few saccules of endoplasmic reticulum and rare vacuoles (Fig. 4A, inset). The nucleus appeared oval and measured approximately 1 μm in its larger diameter. Division of merogonic stages was observed (Figs. 3 and 4): initially, the meront elongated and its nucleus divided by transverse constriction, briefly presenting a binucleated appearance. Afterwards, the cytoplasm constricted, giving rise to two cells identical to the initial meront. Division of the nucleus was sometimes faster than that of the cytoplasm. As a result, on some occasions, it was possible to observe three meronts in a chain (Fig. 4B).

##### *Sporonts* (Fig. 5)

After a few divisions, some meronts underwent morphological transformations to become sporonts. These were ovoid cells, easily recognized by the presence of an electron-dense wall, about 15 nm thick, surrounding the plasma membrane (Fig. 5A, inset). Their smaller diameter (in cross-sections) was about 1 μm, while their larger diameter (in longitudinal sections) was about 2 μm. Initially, endoplasmic reticulum saccules increased in number and dilated, and a few vacuoles appeared (Fig. 5B). These sporonts had a single large central nucleus measuring about 1 μm in diameter. During the nucleus’ division, the sporont became binucleated for a certain time. These dividing sporonts had an elongated shape (measuring up to 5 μm long by 1.5 μm wide). They then constricted in the middle creating two uninucleated sporoblasts (Fig. 6A).

##### *Sporoblasts and sporogenesis* (Fig. 6)

Sporoblasts were uninucleated (Fig. 6A). They were, at first, irregular in shape and then they became ovoid. They were surrounded by a continuous wall about 15–20 nm thick, and were also in direct contact with the cytoplasm of the host cell. Their cytoplasm contained numerous free ribosomes (and polyribosomes) and a few flattened saccules of endoplasmic reticulum (Figs. 6A, 6C, 6D). The appearance of the polar tube was the first obvious manifestation of sporogenesis. At the anterior end of the polar tubule, there was a polar sac containing an electron-opaque material forming an anchoring disc (Fig. 6D). The limiting membrane of the polar sac was continuous with that of the polar tube. Then, the polaroplast appeared to originate from an initially amorphous, electron-dense material associated with the anterior extremity of the polar tube (Figs. 6B, 6C). Within this amorphous mass there were numerous membranes forming parallel saccules that surrounded the manubrium, which is the anterior rectilinear part of the polar tube (Figs. 6C, 6D). In older sporoblasts, the anterior saccules were very flat, while the posterior saccules were dilated, presenting an irregular contour. These saccules originated from a polaroplast made up, in the mature spore, of two distinct parts: anterior lamellar part and posterior granular part. Sporogenesis ended when the initial wall separated from the plasma membrane of the young spore, giving rise to a clear endospore. The initial electron-dense wall became the exospore.

##### *Mature spores* (Figs. 3 and 7–10)

Spores were ovoid and uninucleated, measuring around 2.5 × 1.4 μm. They were surrounded by an exospore with a maximum width around 40 nm and an endospore with a maximum width around 50 nm and about 5 nm wide at the level of the apical part near the anchoring disk (Figs. 7–10). The polar sac was about 30–40 nm wide. The anterior straight part of the polar tube measured about 850 nm long and 115 nm in diameter (Figs. 7B and 9A). The polar tube was isofilar with a diameter of 95–115 nm presenting 8–10 coils arranged in one or, more frequently, in two slightly overlapping rows (Fig. 7A). It was composed of alternate concentric electron-dense and electron lucent layers, with a central tubular element 8 nm in diameter (Fig. 9B). The polaroplast was well-developed and made of a lamellar anterior and external part and a granular internal part (Fig. 8A). Numerous polyribosomes were observed around the polaroplast, nucleus and polar tube (Figs. 7A, 8A and 9B). The nucleus was ovoid, measuring about 650 nm long and 400 nm wide (Fig. 7). The posterior vacuole was irregularly round with a diameter around 375 nm (Fig. 7B).

##### Molecular analyses

One consensus sequence of 1228 bp was obtained (MicroBac 01) for *T. baccigeri* n. gen., n. sp. The results of the BLAST (Supplementary material Table S1) show the highest homologies for *Cucumispora* sp. (MG063425.1) (94.89% sequence identity), *Microsporidium* sp. (HM800853.2) (94.86% sequence identity), and *H. aquatica* (KX364284.1) (94.72% sequence identity).

A final alignment of 1492 bp, including sequences from GenBank, was used for the phylogenetic studies. The results of the Neighbour-Joining (Supplementary material Figure S1) and the Maximum Likelihood (Fig. 1) analyses based on SSU rDNA showed similar results. In both phylogenetic analyses, the novel sequence fell in a well-supported branch together with *Cucumispora* Ovcharenko, Bacela, Wilkinson, Ironside, Rigaud & Wattier, 2010 and several unidentified sequences of crustacean-infecting microsporidia (*Microsporidium* spp.). This branch clustered with the lineage that contained the sequence of *Hyperspora* Stentiford, Ramilo, Abollo, Kerr, Bateman, Feist, Bass & Villalba, 2017. Sequences of two other hyperparasitic microsporidia of the genus *Unikaryon*, also infecting trematodes, fell in another clade of the Superclade V, together with *Dictyocoela* Terry, Smith, Sharpe, Rigaud, Littlewood, Ironside, Rollinson, Bouchon, MacNeil, Dick & Dunn, 2004 and *Triwangia* Wang, Nai, Wang, Solter, Hsu, Wang & Lo, 2013. Overall topology of the tree was consistent with previously published phylogenies [11].

The pairwise distance analyses of the alignment of 16 selected nucleotide sequences (Supplementary material Table S2) support the phylogenetic results, showing that the minimum genetic distance (*p*-distance) was observed between *Toguebayea baccigeri* n. gen., n. sp. and the sequences of Microsporidia sp. EC-2 (KT956053.1) (3.7%) and *Microsporidium* sp. CRANPA (AJ966718.1) (3.9%); being greater than 4.0% for the rest of the analysed sequences, with the maximum genetic distance for *U. panopei* (MW377751.1) (24.2%), *U. legeri* (KX364285.1) (25.2%) and *Potaspora morhaphis* Casal, Matos, Teles-Grilo & Azevedo, 2008 (EU534408.1) (26.8%).

## Discussion

In the present study, we describe the type species of a novel genus of microsporidian, namely *Toguebayea baccigeri* n. gen, n. sp., hyperparasite of the faustulid digenean *Bacciger israelensis*, a parasite of the sparid fish *Boops boops* in the Mediterranean Sea (coast of Tunisia). The description of this new genus and new species is based upon ultrastructural characteristics of its lifecycle, parasitized host, geographical distribution and on sequencing of a partial fragment of the SSU rDNA of this microsporidian hyperparasite.

The phylogenetic analyses indicate that the novel hyperparasite species, *T. baccigeri* n. gen., n. sp., belongs to a new genus of microsporidia, *Toguebayea*, of Clade V (Marinosporidia). Clade V of microsporidia includes three hyperparasite species: *H. aquatica* that parasitises the paramyxid *Marteilia cochillia* [18], *U. legeri* that parasitises the digenean *Parvatrema minutus* (cited as genus *Meiogymnophallus*) parasite of cockles [18], and *U. panopei* that parasitises the digenean *Microphallus* sp., parasite of the crab *Panopeus herbstii* [14]. The molecular analyses highlight that the hyperparasite microsporidian species of the Marinosporidia, *H. aquatica* and *T. baccigeri* n. gen., n. sp. are clearly separated from the subclade of *Unikaryon* hyperparasites *U. legeri* and *U. panopei*.

The representatives of the genera *Cucumispora*, *Hyperspora*, *Myospora* Stentiford, Bateman, Small, Moss, Shields, Reece & Tuck, 2010, *Paradoxium* Stentiford, Ross, Kerr, Bass & Bateman, 2015 and *Thelohania* Henneguy, 1892 that also fell in Clade V share relatively high sequence similarity with the new species; however, they can be differentiated from the latter [10, 18, 19]. In *T. baccigeri* n. gen., n. sp., the sporogony is disporoblastic and all the developmental stages are uninucleated and always in contact with the host cell cytoplasm (epithelial cells of the excretory vesicle) and sporophorous vacuoles are absent. It clearly differs from *Cucumispora dikerogammari* Ovcharenko, Bacela, Wilkinson, Ironside, Rigaud & Wattier, 2010 [10], which presents diplokaryotic meronts, sporonts and mature spores (see Table 1). In *Paradoxium irvingi* Stentiford, Ross, Kerr, Bass & Bateman, 2015 all stages are also in close contact with the host cell cytoplasm, but this genus differs from the new genus *Toguebayea* in the formation of binucleated and later tetranucleated meronts during merogony [19]. In *Thelohania butleri* Johnston, Vernick & Sprague, 1978, the most evident difference is the presence of groups of eight spores enclosed in sporophorous vacuoles [2]. In *Myospora metanephrops* Stentiford, Bateman, Small, Moss, Shields, Reece & Tuck, 2010, a myosporid microsporidian closely related to *T. butleri* (see Fig. 1), all lifecycle stages (meronts, sporonts, sporoblasts and spores) are diplokaryotic. Moreover, both merogony and sporogony are clearly different from those of *T. baccigeri* n. gen., n. sp. Thus, in *M. metanephrops* diplokaryotic meronts divide into tetranucleated meronts (with two diplokaryotic nucleus) and later into octonucleated meronts (with four diplokaryotic nucleus), and tetranucleated sporonts (with two diplokaryotic nucleus) form two diplokaryotic sporoblasts [17]. Finally, *H. aquatica* shows the most similar development with *T. baccigeri* n. gen., n. sp.; all the developmental stages are uninucleated and in close contact with the host cell cytoplasm. The most evident morphological differences are the size of the mature spores (1.2 × 1.0 μm in *H. aquatica* vs. 2.5 × 1.4 μm *T. baccigeri* n. gen., n. sp.) and the number of polar tube coils (4 in *H. aquatica* vs. 8 to 10 in *T. baccigeri* n. gen., n. sp.) [18] (see Table 1). Traditional taxonomy based on morphological features and the molecular SSU rDNA-based phylogenetic analyses show great discrepancies, and this fact has been emphasized by several authors [11, 17, 24]. For example, Vossbrinck and Debrunner-Vossbrinck [24] highlighted the importance of ecological trends, such as the parasitised host and its habitat, in the molecular phylogenetic grouping of the Microsporidia. Clearly, the use of these ecological characteristics is more consistent with evolutionary relationships of microsporidia than traditional classification based on morphological and lifecycle features. According to these authors, the clade Marinosporidia includes microsporidia parasitising predominantly marine hosts, with a few exceptions inhabiting the freshwater environment [2, 10, 17, 19]. All species mentioned above are parasites of marine and freshwater crustaceans (Decapoda and Amphipoda), with only one exception: *Hyperspora*, which is a hyperparasite of *M. collichia* (Paramyxida) in the marine mollusc *Cerastoderma edule* in Ría de Arousa [18]. *Toguebayea baccigeri* n. gen., n. sp. is strikingly different in this respect: it is a hyperparasite of the adult stage of a digenean trematode parasitising a marine fish.

As to the geographical distribution, only the new species *T. baccigeri* was found in the Mediterranean Sea, off Salakta (Tunisia). All other species of Clade V discussed above were found in freshwater environments, such as ponds, lakes, reservoirs, oases or rivers in continental areas, or in other marine areas (North Sea, coast of Galicia (Spain), Pacific coast of Canada and New Zealand) (Table 1).

Thus, ultrastructure of developmental stages, the host species, habitat, geographical distribution, and phylogenetic position within Clade V identify the discovered microsporidium as a new species of a new genus. Our study also confirms for the first time microsporidian infection in a digenean of the Faustulidae family and adds a new genus and species, *Toguebayea baccigeri*, to the list of hyperparasitic microsporidia.

## Conflict of interest

The authors declare that they have no conflict of interest.

## Supplementary Materials

The supplementary materials of this article are available at https://www.parasite-journal.org/10.1051/parasite/2022007/olm.**Figure S1.**
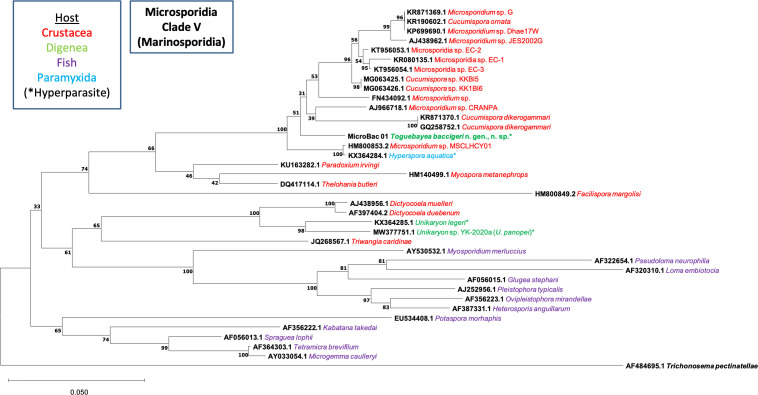
Phylogenetic analysis, based on the Neighbour-Joining method, of Microsporidia sequences based on the SSU rDNA gene. *Trichonosema pectinatellae* (AF484695.1) was used as the outgroup. MicroBac01 corresponds to the sequence obtained for *Toguebayea baccigeri* n. gen., n. sp.Supplementary Tables*Table S1*. Result of the BLAST analyses for the nucleotide sequence obtained in this study MicroBac 01. (QC: query cover; Idt: identity).*Table S2*. Comparison of small subunit gene (SSU rDNA) sequences among Microsporidia parasites. Pairwise distance (bottom diagonal) obtained by p-distance.
