# Supplementary material for: Ultrastructural and molecular study of the microsporidian Toguebayea baccigeri n. gen., n. sp., a hyperparasite of the digenean trematode Bacciger israelensis (Faustulidae), a parasite of Boops boops (Teleostei, Sparidae)
Source: Parasite. 2022 Feb 1;29:2. doi: 10.1051/parasite/2022007 (PMC8805611; doi:10.1051/parasite/2022007)
Supplement: Supplementary Tables — Table S1. Result of the BLAST analyses for the nucleotide sequence obtained in this study MicroBac 01. (QC: query cover; Idt: identity). Table S2. Comparison of small subunit gene (SSU rDNA) sequences among Microsporidia parasites. Pairwise distance (bottom diagonal) obtained by p-distance. [file parasite-29-2-s1.pdf]

Table S1. Result of the BLAST analyses for the nucleotide sequence obtained in this study MicroBac 01. (QC: query cover; Idt: identity).

**Microsporidia**	**Acc. Number**	**Host**	**Taxonomy**	**QC**	**Idt**
Cucumispora sp. KKBl5	MG063425.1	*Brandtia latissima*	Crustacea	99%	94.89%
Cucumispora sp. KK1Bl6	MG063426.1	*Brandtia latissima*	Crustacea	99%	94.81%
Microsporidium sp. MSCLHCY01	HM800853.2	*Lepeophtheirus hospitalis*	Crustacea	99%	94.86%
Hyperspora aquatica	KX364284.1	*Marteilia cochillia*	Paramyxida	99%	94.72%
Microsporidium sp. Dhae17W	KP699690.1	*Dikerogammarus haemobaphes*	Crustacea	99%	94.01%
Microsporidium sp. 1049	FN434092.1	*Gammarus duebeniduebeni*	Crustacea	99%	93.93%
Microsporidium sp. JES2002G	AJ438962.1	*Gammarus chevreuxi*	Crustacea	99%	93.49%
Cucumispora ornata	KR190602.1	*Dikerogammarus haemobaphes*	Crustacea	94%	93.58%
Cucumispora dikerogammari	GQ426188.1	*Dikerogammarus villosus*	Crustacea	99%	91.86%
Cucumispora dikerogammari	GQ258752.1	*Dikerogammarus villosus*	Crustacea	99%	91.85%
Cucumispora dikerogammari<br>(in GenBank: *Microsporidium* sp. RW-2009a)	KR871370.1	*Dikerogammarus haemobaphes*<br>*Dikerogammarus villosus*	Crustacea	76%	91.97%
Microsporidium sp. CRANPA	AJ966718.1	*Crangonyx pseudogracilis*	Crustacea	65%	94.89%
Microsporidium sp. EC-3	KT956054.1	*Eulimnogammarus cyaneus*	Crustacea	69%	94.60%
Microsporidium sp. EC-1	KR080135.1	*Eulimnogammarus cyaneus*	Crustacea	69%	94.50%
Microsporidium sp. EC-2	KT956053.1	*Eulimnogammarus cyaneus*	Crustacea	68%	94.94%
Microsporidium sp. G	KR871369.1	*Dikerogammarus haemobaphes*<br>*Gammarus pulex*<br>*Gammarus roeseli*	Crustacea	76%	94.37%

Table S2. Comparison of small subunit gene (SSU rDNA) sequences among microsporidia parasites. Pairwise distance (bottom diagonal) obtained by p-distance.

Species (GenBank Acc. Number)	1	2	3	4	5	6	7	8	9	10	11	12	13	14	15	16
1. Microsporidia sp. EC-1 (KR080135.1)	-															
2. Microsporidia sp. EC-2 (KT956053.1)	0.006	-														
3. *Microsporidium* sp. (FN434092.1)	0.040	0.035	-													
4. *Microsporidium* sp. CRANPA (AJ966718.1)	0.053	0.047	0.043	-												
5. *Microsporidium* sp. Dhae17W (KP699690.1)	0.023	0.017	0.049	0.049	-											
6. *Thelohania butleri* (DQ417114.1)	0.134	0.128	0.118	0.116	0.137	-										
7. *Potaspora morhaphis* (EU534408.1)	0.274	0.266	0.268	0.274	0.263	0.238	-									
8. *Toguebayea baccigeri* (MZ413057)	0.043	0.037	0.043	0.039	0.049	0.114	0.268	-								
9. *Cucumispora* sp. KKBl5 (MG063425.1)	0.009	0.004	0.035	0.052	0.021	0.125	0.266	0.041	-							
10. *Cucumispora* sp. KK1Bl6 (MG063426.1)	0.009	0.004	0.035	0.051	0.021	0.125	0.266	0.041	0.000	-						
11. *Microsporidium* sp. MSCLHCY01 (HM800853.2)	0.057	0.051	0.053	0.047	0.063	0.120	0.277	0.051	0.049	0.049	-					
12. *Hyperspora aquatica* (KX364284.1)	0.055	0.049	0.055	0.049	0.065	0.123	0.274	0.053	0.047	0.047	0.002	-				
13. *Unikaryon legeri* (KX364285.1)	0.263	0.255	0.267	0.246	0.258	0.230	0.250	0.252	0.255	0.255	0.239	0.239	-			
14. *Unikaryon panopei* (MW377751.1)	0.260	0.253	0.259	0.242	0.255	0.233	0.251	0.242	0.253	0.253	0.234	0.234	0.051	-		
15. *Cucumispora ornata* (KR190602.1)	0.023	0.017	0.049	0.049	0.000	0.137	0.263	0.049	0.021	0.021	0.063	0.065	0.258	0.255	-	
16. *Cucumispora dikerogammari* (GQ258752.1)	0.067	0.061	0.078	0.055	0.070	0.146	0.277	0.067	0.065	0.065	0.078	0.076	0.266	0.267	0.070	-
